# In Situ X-Ray Imaging and Machine Learning in Ultrasonic Field-Assisted Laser-Based Additive Manufacturing: A Review

**DOI:** 10.3390/ma19061227

**Published:** 2026-03-20

**Authors:** Zhihao Fu, Yu Weng, Zhian Deng, Jie Pan, Ao Li, Ling Qin, Gang Wu

**Affiliations:** 1 College of Pipeline Engineering, Xi’an Shiyou University, Xi’an 710065, China; 20211010030@stumail.xsyu.edu.cn (Z.F.); jackpan@xsyu.edu.cn (J.P.); wugang@xsyu.edu.cn (G.W.); 2 School of Energy, Quanzhou Vocational and Technical University, Quanzhou 362000, China; dza--062826@163.com; 3 Department of Materials Science and Engineering, University of California, Irvine, CA 92697, USA; aol8@uci.edu; 4 Center of Innovation for Flow Through Porous Media, Department of Energy and Petroleum Engineering, University of Wyoming, Laramie, WY 82071, USA

**Keywords:** ultrasonic-assisted additive manufacturing, synchrotron X-ray imaging, in situ monitoring, keyhole dynamics, porosity prediction, image-recognition machine learning, physics-informed machine learning

## Abstract

Metal additive manufacturing (AM) offers unprecedented opportunities to fabricate complex, lightweight metallic components, yet its practical deployment remains fundamentally constrained by defects arising from rapid melting and solidification. Cyclic thermal transients generate cracks, pores, residual stresses, and lack-of-fusion regions, undermining mechanical performance and reliability. Ultrasonic field-assisted laser-based additive manufacturing (UF-LBAM) has emerged as a powerful approach to manipulate melt pool dynamics and suppress defect formation. Nevertheless, the governing physical mechanisms remain poorly understood, particularly under highly non-equilibrium ultrasonic excitation, where acoustic pressure oscillations, melt convection, cavitation, and solidification are intricately coupled across multiple temporal and spatial scales. Here, we provide a systematic review of X-ray based fundamental studies in UF-LBAM and the diverse applications of machine learning (ML), detailing the literature selection criteria and methodology. We highlight advances spanning synchrotron X-ray revealed physical phenomena, ML-driven real-time monitoring and defect prediction, and pathways toward industrial implementation. Critical challenges persist, including fundamental physics gaps, transferability of ML models across alloy systems, and real-time control limitations. We further identify promising directions for the field, such as physics-informed models, multimodal diagnostics, and closed-loop control, which together promise to unlock the full potential of UF-LBAM for high-performance metal component fabrication.

## 1. Introduction

Metal additive manufacturing (AM) has emerged as a transformative advanced manufacturing paradigm, enabling the fabrication of lightweight components with highly complex geometries and unprecedented design freedom, while offering substantial economic potential [1,2,3,4]. Its adoption has rapidly expanded across aerospace, automotive, and oil-and-gas industries, where performance-critical and geometrically sophisticated components are required. For example, rocket engine nozzles, combustion chambers, and heat exchangers with complex internal cooling channels were produced using LBAM by SpaceX. In aerospace, topologically optimized structural supports were also fabricated using this approach, enabling single-piece production of complex geometries that reduce weight and part count. Among the various metal AM technologies, laser-based additive manufacturing (LBAM) is particularly prominent owing to its capability to realize intricate architectures and to support disruptive engineering applications, thereby progressively reshaping conventional manufacturing routes [5]. LBAM operates by employing a focused laser beam to selectively melt, sinter, or consolidate metallic feedstocks—typically in powder, wire, or liquid form—through a layer-by-layer deposition process [6]. Depending on the feedstock delivery method, LBAM encompasses powder-blown [7], powder-bed fusion [8], and wire-fed techniques [9]. Powder-blown techniques, commonly referred to as directed energy deposition (DED), feed metal powder through a nozzle directly into a melt pool generated by a laser or electron beam. This enables layer-by-layer deposition at relatively high build rates, making it particularly well-suited for large-scale fabrication and component repair. Powder-bed fusion (PBF) involves spreading a thin layer of powder across the build platform, followed by selective melting with a focused laser or electron beam. This approach excels at producing complex three-dimensional geometries with superior dimensional accuracy and surface finish. Wire-fed processes utilize metallic wire as feedstock, which is continuously fed and melted by an energy source—such as an electric arc, laser, or electron beam—before being deposited onto a substrate or previous layer. These methods offer excellent material utilization (near 100%) and high deposition efficiency, rendering them ideal for manufacturing large metallic components.

Despite its remarkable advantages, LBAM remains intrinsically susceptible to process variability and instability [10], which pose significant challenges to achieving consistent part quality. These challenges originate from strongly coupled multiphysics interactions involving laser-matter energy absorption, vaporization and recondensation, melt pool dynamics, and microstructural evolution [11,12]. These phenomena frequently result in defects such as cracks, porosity, distortion, and lack-of-fusion, which compromise the mechanical properties and functional reliability of the manufactured parts. Such defects are governed not only by material properties and part geometry, but also by processing parameters (e.g., laser power, scan speed, and beam diameter) and are further exacerbated by stochastic fluctuations inherent to the process [13,14].

To mitigate solidification defects and improve process stability and part quality, underlying defect formation mechanisms have been widely investigated [15], and external field-assisted approaches have recently gained attention [16,17]. Among these, UF-LBAM is particularly promising due to its pronounced grain-refinement effect within the melt pool [18,19]. Ultrasonic excitation enables precise modulation of melt pool convection, solidification dynamics, and microstructural evolution [20,21]. For instance, ultrasonic vibrations can enhance melt pool stirring, suppress porosity and inclusions, and promote densification and microstructural uniformity. Nevertheless, the underlying mechanisms remain poorly understood, largely because the inherent opacity of metal or alloy precludes direct in situ observation.

For more than a century, X-ray imaging has been instrumental in probing the internal structures of opaque materials [22]. Since the emergence of third-generation synchrotron facilities in the 1990s, advances in synchrotron-based X-ray imaging have been widely adopted across diverse areas of materials research [23]. The high-brightness X-ray beams and advanced detector technologies available at synchrotron sources enable real-time, in situ investigations of LBAM processes [15,24]. These techniques can capture real-time melt pool dynamics, defect initiation, and phase transformation kinetics, offering new insights into the understanding of highly dynamic manufacturing processes.

With the massive amount of data generated by X-ray-based radiography and tomography (capturing internal microstructural dynamics), thermal imaging (recording surface temperature evolution), and optical microscopy (providing in situ observation), manual processing and analysis of these datasets have become virtually impossible in terms of both time and accuracy, necessitating the adoption of advanced computational tools for efficient feature extraction, defect identification, and predictive modeling of process dynamics. Machine learning (ML) has therefore emerged as a powerful tool for monitoring thermal imaging data, extracting information from high-dimensional X-ray datasets, and providing predictive insights into process dynamics and defect formation [25,26,27]. Models such as k-nearest neighbor algorithms [28], enable efficient segmentation and quantification of in situ images for melt pool characterization, while convolutional neural networks (CNNs) accelerate X-ray computed tomography (XCT) analysis, allowing high-precision detection of solidification defects such as pores and cracks [29]. ML-driven reconstruction algorithms, combined with CAD models, facilitate rapid, high-resolution XCT data acquisition suitable for industrial LBAM [30]. Moreover, CNNs can predict melt pool behavior and defect evolution in real time, providing valuable feedback for external field-assisted processes [31,32].

Compared with previous reviews, this review stands out by emphasizing three emerging directions: (i) innovative strategies for ultrasound field-assisted LBAM, with particular focus on its deep integration with laser powder bed fusion (LPBF), directed energy deposition (DED), and wire arc additive manufacturing (WAAM); (ii) fundamental physical insights revealed through in situ synchrotron imaging; and (iii) ML applications in AM, highlighting their critical role in high-precision image processing and industrial process optimization. This review systematically summarizes recent advances in ultrasound-assisted LBAM, emphasizing the pivotal influence of ultrasound on keyhole dynamics, melt pool behavior, grain refinement, and the formation of solidification defects. It further analyzes state-of-the-art ML approaches for in situ thermal imaging and X-ray based characterization, including XCT data processing, automated defect identification, and quantitative analysis, underscoring their potential for real-time monitoring and predictive process control. Finally, the review provides an in-depth discussion of industrial prospects, technical bottlenecks, and future directions for the integration of field-assisted LBAM with ML, aiming to enable the fabrication of high-performance metallic structures, intelligent manufacturing workflows, and next-generation additive manufacturing strategies.

## 2. The Fundamental Issues of Ultrasonic-Assisted Metal Additive Manufacturing

A fundamental challenge in UF-LBAM lies in the opacity of metals or alloys, which limits real-time observation of the underlying physical mechanisms when ultrasound is applied during LBAM. At the microscale, the nucleation mechanisms of metallic phases under ultrasonic excitation in LBAM remain poorly understood [33]. At the mesoscale, ultrasonic bubble nucleation, growth, oscillation, and collapse, tightly coupled with acoustic streaming and the evolving solidification front, interact in highly complex and non-linear ways, severely limiting the ability to quantitatively link these phenomena to microstructural evolution [34]. At the macroscale, the absence of direct measurements of melt pool dynamics and keyhole stability further constrains the optimization of ultrasonic parameters and impedes mechanistic understanding and precise control over solidification defect formation [35]. Consequently, researchers primarily rely on post-process analysis [36], acoustic emission [37], or molecular dynamics and numerical simulations [38], all of which are constrained by resolution and modeling assumptions. While numerical models provide insights into melt pool dynamics, cavitation behavior, and microstructural evolution, their accuracy remains limited without experimental validation. The transient and coupled interactions among bubbles, acoustic streaming, and the solidification front are challenging to fully capture in simulations, and oversimplified assumptions or limited resolution can result in discrepancies between predicted and actual outcomes.

In this work, the literature survey was conducted using major scientific databases, including Web of Science, Scopus, and Google Scholar. Publications from 2000 to 2025 were considered, with particular emphasis on studies published after 2015 due to the rapid development of in situ X-ray imaging and machine learning techniques in metal additive manufacturing. This period marks the rapid development and maturation of metal additive manufacturing, driven by advances in laser- and electron-beam processing, improved process monitoring and control, and a deeper understanding of microstructural evolution and defect formation, as well as a significant increase in high-quality experimental and computational studies and a large number of related publications. This timeframe captures the transition of AM from proof-of-concept demonstrations toward industrial-scale applications in aerospace, automotive, and energy sectors, providing a rich and relevant dataset for reviewing current challenges and research trends. The selection criteria focused on peer-reviewed journal articles and high-impact conference proceedings directly related to ultrasonic-assisted additive manufacturing, in situ synchrotron or laboratory-based X-ray characterization, and data-driven modeling approaches. Review articles were included to provide contextual background, while purely theoretical studies lacking experimental relevance to LBAM were excluded.

## 3. Scientific Insights from X-Ray Studies on UF-LBAM

### 3.1. Ultrasonic Loading Modes

**Figure 1:**
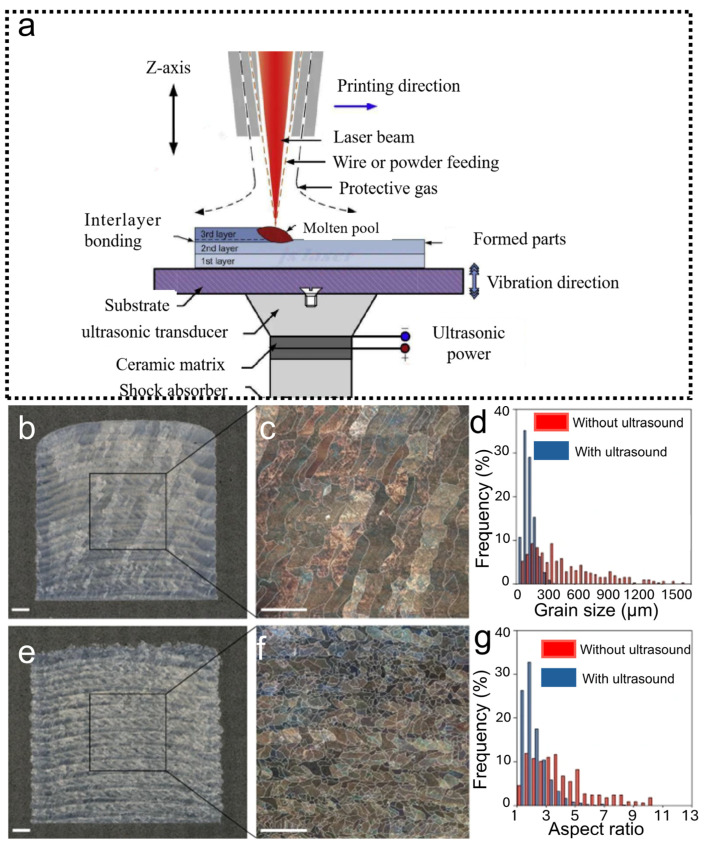
(**a**) Schematic of the experimental setup [42]. (**b**,**e**) Optical microscopy images of samples without (**b**) and with (**e**) ultrasound. (**c**,**f**) Polarized light microscopy images showing large columnar grains (**c**) and fine equiaxed grains (**f**). (**d**,**g**) Histograms of prior-β grain size (**d**) and prior-β grain aspect ratio (**g**) measured from traced prior-β grain images for samples without and with ultrasound [21]. Scale bars, 1 mm.

**Figure 2:**
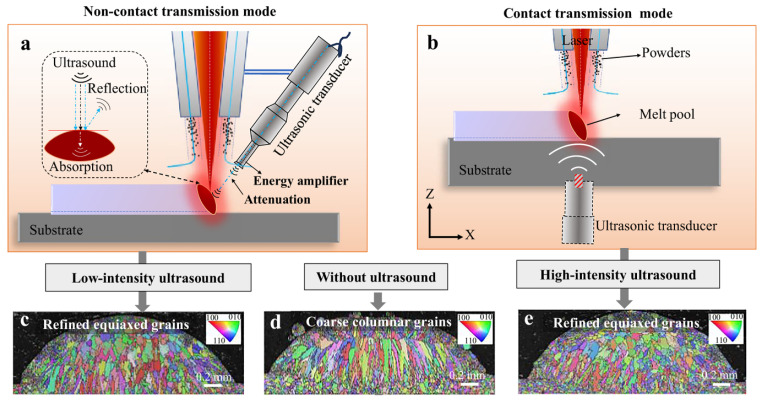
Ultrasound-assisted laser melting and deposition. (**a**) Schematic of low-intensity ultrasound in a non-contact transmission mode. (**b**) Schematic of high-intensity ultrasound in a contact transmission mode. Grain structure of a single deposited track under low-intensity ultrasound (**c**), without ultrasound (**d**), and high-intensity ultrasound (**e**) [43].

Until now, UF-LBAM was generally realized through two complementary approaches. In the early development of UF-LBAM, one approach relied on direct-contact transmission, in which ultrasound was coupled to the melt pool through a solid substrate to maximize acoustic intensity, with the ultrasonic transducer positioned in physical contact with the baseplate [21,39] (Figure 1a and Figure 2b). Figure 1b–g reveal a pronounced microstructural difference in AM-fabricated Ti-6Al-4V samples. Without ultrasound, the material exhibits long (~millimeter-scale), ~0.5 mm-wide columnar prior-β grains that span multiple deposited layers. In contrast, ultrasound application yields fine (~100 µm) equiaxed grains, dramatically increasing prior-β grain number density from 3.3 mm^−2^ to 65.0 mm^−2^—clear evidence of enhanced nucleation during solidification. This shift also markedly improves the uniformity of grain size and aspect ratio distributions. However, contact-transmission continuous approaches face significant challenges in achieving uniform grain refinement, particularly for large or geometrically complex parts [21]. Variations in the distance between the melt pool and the ultrasonic source, especially beyond the third deposited layer, lead to substantial fluctuations in ultrasonic amplitude within the melt pool, making it difficult to maintain consistent ultrasonic effects. Consequently, layer-by-layer fabrication produces inconsistent and unpredictable ultrasonic effects, resulting in heterogeneous solidified microstructures and mechanical properties. This inhomogeneity remains a key limitation of current high-intensity UF-LBAM.

Pulsed ultrasonic loading is often preferred in industrial applications and offers distinct advantages over continuous or fixed-frequency ultrasound [40]. Pulsed ultrasound can maintain the peak pressures required for effective dendrite fragmentation and grain refinement while intermittently delivering energy, thereby reducing heat accumulation in the transducer, lowering the risk of overheating, and extending equipment lifetime. This intermittent application also optimizes energy utilization, preventing excessive cavitation that may cause melt pool surface damage or flow instabilities under continuous ultrasound. Moreover, it allows precise control over pulse duration (e.g., 10–50 ms) and intervals (e.g., 100–500 ms), enabling targeted modulation of melt pool dynamics, improving process stability, and reducing overall energy consumption by approximately 20–30%, particularly in long-duration, high-intensity manufacturing scenarios [41].

For example, in wire arc AM of high-strength 2024 Al, ultrasonic frequency pulsed arcs (20–40 kHz, pulse cycles synchronized with the arc period) were applied to assist the deposition of thin-walled structures. This approach markedly refined grain structures and reduced porosity. Compared with continuous ultrasound, microstructural uniformity was improved, microhardness uniformity increased by 15–25%, and vertical tensile properties were enhanced by ~20%, leading to improved isotropy in heat-treated components. Such strategies are especially advantageous for the fabrication of complex, multilayer aerospace lightweight frameworks.

To address these limitations, a recently developed non-contact transmission mode was designed, enabling ultrasound to be applied to the melt pool without physical contact with the baseplate [43]. Specifically, a low-intensity, non-contact UAMAM approach was developed to stabilize ultrasonic input into the melt pool, enable uniform grain refinement, and examine the effects of acoustic streaming on the laser melting process (Figure 2a). A key feature of this method is its synchronous operation with the laser, ensuring coordinated ultrasonic assistance throughout the melting and solidification stages. The EBSD results show that this approach effectively refines grains and reduces fusion defects during the layer-by-layer fabrication of large samples, achieving uniform microstructures in Figure 2c–e, and overcoming critical limitations of existing ultrasound-assisted AM techniques. Grain refinement greatly improves fatigue performance and resistance to crack propagation.

Under cyclic loading, coarse columnar grains often promote long-range slip bands along grain boundaries or within grains, leading to early crack initiation. In contrast, fine equiaxed grains distribute stress more uniformly, reducing local stress concentrations and extending the crack initiation phase of fatigue life. Fine-grained structures also exhibit superior resistance to environmentally assisted cracking mechanisms, such as hydrogen embrittlement and stress corrosion cracking. The increased grain boundary area disperses hydrogen segregation and impedes rapid crack advance along preferential paths. These combined benefits make grain refinement a critical strategy in modern metal additive manufacturing (e.g., laser cladding, electron beam melting) as well as traditional forging and heat-treatment processes, delivering particularly substantial gains in comprehensive mechanical performance for difficult-to-deform alloys like Ti-6Al-4V and Inconel 718. Moreover, the main distinction between non-contact and contact transmission modes is reflected in the effective ultrasonic intensity delivered to the melt pool. The energy transmitted into the molten region is determined by acoustic transmission across multiple interfaces. The acoustic impedance of the transducer, amplitude rod, substrate, and molten metal dictates the proportion of energy that is reflected versus transmitted. When impedance matching is poor, the energy reaching the melt pool is reduced, limiting cavitation and acoustic streaming; conversely, when matching is optimized, dendrite fragmentation and non-spontaneous nucleation are efficiently induced. For instance, in Ti alloys, which possess relatively high acoustic impedance (~27 MRayl), higher input power or tailored coupling strategies are generally required to achieve effective cavitation. In contrast, in aluminum alloys (~17 MRayl), strong melt pool agitation and columnar-to-equiaxed transitions can be realized at lower power levels. Therefore, the understanding and optimization of impedance matching are considered critical for achieving consistent microstructural refinement across different material systems [44].

### 3.2. Melt Pool and Keyhole Dynamics During UF-LBAM

In LBAM, several key physical phenomena occur during the process, as summarized in Table 1. Among them, the melt pool and keyhole induced by the laser play a central role, directly influencing microstructural evolution, defect formation, and the final quality of deposited material. The size, morphology, and dynamic behavior of the melt pool and keyhole govern solidification rates, grain structure, and residual stress distributions within individual layers. Precise control of melt pool characteristics is particularly critical during multi-layer deposition, where cumulative thermal effects can induce distortion, porosity, or microstructural heterogeneity. Therefore, direct observation and control of melt pool dynamics are essential for producing high-density, defect-free components with reproducible mechanical properties.

**Figure 3:**
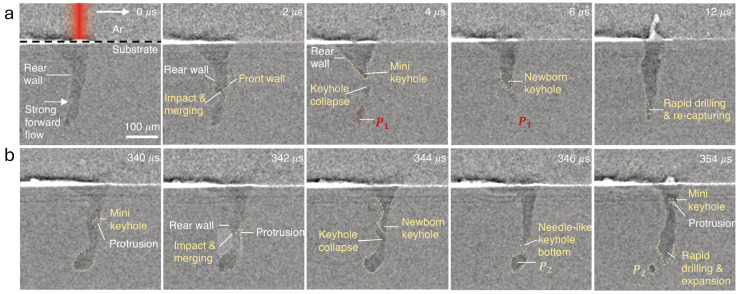
Megahertz X-ray images capturing (**a**) intrinsic keyhole oscillation without pore formation and (**b**) perturbative keyhole oscillation with keyhole pore formation [45]. The dashed lines highlight the keyhole.

Ren et al. [45] performed operando experiments at the 32-ID-B beamline of the Advanced Photon Source, Argonne National Laboratory (Lemont, IL, USA) (Figure 3a). High-energy X-rays penetrated single-layer powder beds or bare substrates, revealing subsurface structural dynamics, while a thermal camera captured an angled top view of the melt pool. A continuous-wave fiber laser scanned the samples along a single line at varying powers and speeds. Figure 3 show two representative behaviors: intrinsic keyhole oscillation without pore formation and perturbative keyhole oscillation that produces a keyhole pore. A similar keyhole phenomenon was also reported by K. Schricker et al. in laser beam welding [46]. Coherent HighLight FL8000-ARM fiber laser (Santa Clara, CA, USA), producing a concentric core–ring beam at a wavelength of 1070 nm, was used. With the selected fiber configuration and optical system, the beam parameters at the focal plane were core diameter 89 μm, inner ring 109 μm, and outer ring 208 μm. Preliminary tests confirmed that the laser penetration depth remained within the field of view of the imaging system. Experiments were therefore conducted at a maximum total power of 3.5 kW with a constant scanning speed. The significance of these two representative studies lies in establishing a direct link between processing parameters and keyhole morphology through direct experimental evidence.

**Figure 4:**
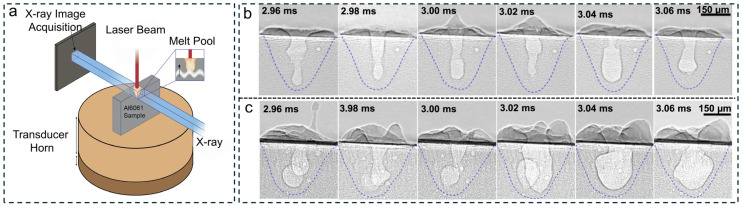
(**a**) Schematic diagram illustrating the experimental setup for high-speed X-ray imaging of melt pools on a vibrating substrate. X-ray image sequences showing laser-generated molten Al6061 pools (**b**) without and (**c**) with ultrasound [47].

To systematically investigate the effect of ultrasound on melt pool morphology, L. Mutswatiwa et al. [47] performed ultrafast synchrotron X-ray imaging experiments at the 32-ID-B beamline of the Advanced Photon Source (see Figure 4a). The setup comprised a continuous-wave ytterbium fiber laser (power range 100–560 W), a high-speed X-ray imaging system, and an Al6061 sample mounted vertically on a Langevin transducer driven at its lowest-order extensional resonance frequency of 20.2 kHz. Single-pulse X-ray images were acquired at 50 kHz, enabling real-time capture of melt pool dynamics, cavitation bubble motion, and solidification processes with unprecedented temporal and spatial resolution. Such experimental configuration allowed for direct visualization of transient phenomena that are otherwise hidden in opaque metals, such as keyhole fluctuations, bubble nucleation, growth, motion, and interactions with the solidification front. Figure 4b,c show X-ray image sequences of a stationary laser-generated Al6061 melt pool without and with ultrasonic excitation, respectively.

Without ultrasound, a narrow and deep keyhole forms in the melt pool, whose tip occasionally pinches off to generate bubbles, leading to porosity. These bubbles generally settle at the bottom of the melt pool, where they are rapidly trapped by the advancing solidification front, forming voids. Pronounced fluctuations in keyhole depth were also observed, indicating the inherent instability of the keyhole structure and suggesting that uncontrolled keyhole dynamics are a major source of defects in LBAM [48]. Synchrotron X-ray imaging allowed direct, real-time visualization of melt pool morphology under ultrasound and bubble dynamics in the melt pool, providing clear evidence of its role in stabilizing the melt pool. These findings demonstrate the power of combining ultrafast synchrotron X-ray imaging with AM to reveal the fundamental mechanisms controlling keyhole and melt pool behavior, offering guidance for process optimization and the development of more reliable AM strategies.

### 3.3. Cavitation Bubble Dynamics and Solid-Phase Interactions in the Melt Pool

Early experiments have shown that when ultrasound is applied, ultrasonic excitation can induce the nucleation, growth, and collapse of cavitation bubbles within the melt pool, generating high-pressure shock waves, microjets, and intense flow perturbations that not only enhance convection and solute transport to promote grain refinement, but also, through coupling with solid/semi-solid interfaces, strongly influence the formation and evolution of porosity, lack-of-fusion defects, and inclusions [49].

**Figure 5:**
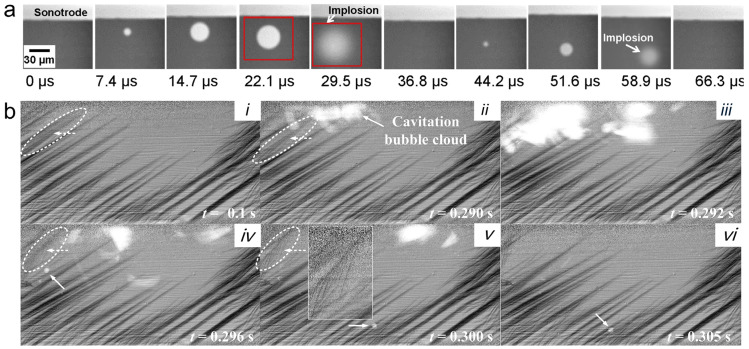
(**a**) An X-ray image sequence acquired using 135,780 fps at Sector 32-ID-B of APS, showing the bubble implosion immediately below the sonotrode tip in the Bi-8% Zn alloy at 427 °C; the input ultrasonic intensity was 276 W/mm^2^ [52]. (**b**) Images of a passing cavitation bubble cloud breaking up the primary Al_2_Cu intermetallic dendrites at the top corner of the field of view [53]. The fragments were then swept out of the field of view by acoustic streaming flow at different times: (i) 0.10 s, (ii) 0.29 s, (iii) 0.292 s, (iv) 0.296 s, (v) 0.300 s, and (vi) 0.305 s.

Figure 5a shows the oscillation and collapse of cavitation bubbles in liquid melt, which cannot be captured by conventional characterization techniques, captured via ultrafast synchrotron X-ray phase-contrast imaging at the 32-ID-B beamline of the Advanced Photon Source. Due to the markedly different physical properties of liquid metal compared with other liquids, although cavitation bubble dynamics have been previously observed in water, this work provides the first direct observation of bubbles in a liquid metal melt undergoing the complete lifecycle—from nucleation and growth to collapse. Moreover, Figure 5b reveals similar bubble dynamics alongside fragmentation and detachment of primary Al_2_Cu intermetallic phases after ultrasound application. Transient cavitation bubble clouds directly drove these events, entering the field of view from the upper left and exiting toward the upper right, impacting Al_2_Cu phases and causing their breakage. A representative dendrite (dashed arrows, circled in Figure 5(bi–bvi)) fractured and rotated under the bubble cloud, while the resulting fragments retained characteristic dendritic morphology, including intact primary trunks and secondary arms (see Figure 5(bvi)). Morphologically preserved dendrite fragments of this type were widespread throughout the microstructure after ultrasound application. Fragmentation events very closely tracked cavitation bubble dynamics and highly depended on ultrasonic intensity. At moderate intensities, high-frequency bubble oscillations dominated, promoting dendrite breakage through sustained acoustic streaming and microjets. At higher intensities, violent bubble implosions generate strong shock waves, releasing more energy and causing more extensive fragmentation. These results provide direct evidence that the interplay between cavitation and ultrasonic intensity governs microstructural refinement after ultrasound application. Under extreme non-equilibrium solidification in LPBF, local melt pool temperature gradients—typically 5–20 K/μm (5 × 10^6^–2 × 10^7^ K/m), with in situ values often 1.6–8.7 K/μm at the front and bottom—directly impose spatial selectivity on cavitation activity [50]. In the high-temperature core (peak temperatures 3000–5000 K, exceeding boiling points and reaching 3300–3700 K in Ti-6Al-4V and similar alloys), dynamic viscosity drops by several times to an order of magnitude relative to edges, while surface tension decreases markedly (dσ/dT ≈ −(1–4) × 10^−4^ N/(m·K), driving outward Marangoni flow). This reduces bubble expansion resistance and, via an exponential rise in saturated vapor pressure, substantially lowers the cavitation threshold, enabling bubbles in the hot zone to overcome nucleation barriers more easily and generate intense microjets through nonlinear oscillations (local velocities up to ~8 m/s in vortices/jets). However, this thermal enhancement is highly transient: rapid laser scanning (0.5–2 m/s) yields cooling rates of 10^5^–10^6^ K/s (measured maxima 1–40 K/μs or 10^6^–4 × 10^7^ K/s, with ranges like 6.5–56 K/μs in Ti-6Al-4V), causing abrupt viscosity increase and cavitation quenching. Thus, the cavitation field acts as a dynamic “tracking field” aligned with the evolving thermal landscape (localized to the low-viscosity core and following the laser path), with spatial energy dissipation heterogeneity fundamentally driving microstructural refinement variations (e.g., cell/grain sizes from hundreds of nm to several μm depending on local gradients and rates) [51].

In situ X-ray imaging also enables the real-time observation of cavitation bubbles and melt pool oscillations induced by ultrasound, providing a high-resolution window into transient multiphysics phenomena. Such observations serve as a critical validation dataset for numerical models, enhancing their predictive capability. For example, Qin et al. [54,55] tracked the nucleation, growth, coalescence, and collapse of cavitation bubbles in different liquid media in real time, using X-ray data to calibrate model parameters with high precision, including surface tension, viscosity, and bubble dynamics. Based on this calibration, the models can accurately assess the effects of ultrasound during solidification, including local stresses induced by bubble collapse on the solid and the mechanical response of dendrites, providing a reliable framework for understanding microstructure evolution and defect formation mechanisms during AM.

### 3.4. Ultrasound Excitation Frequency During AM

In industrial applications, fixed-frequency ultrasound (e.g., 20 kHz) carries a high risk of generating standing wave patterns during AM, which prevents uniform grain refinement in large, complex parts [56,57]. Such behavior stems from the formation of stationary standing-wave patterns under fixed-frequency excitation, which generate persistent nodes and antinodes in the melt pool, thereby causing heterogeneous acoustic energy distribution and spatially non-uniform cavitation activity. As a solution, multi-frequency modulated ultrasound has been proposed to overcome these limitations. Multi-frequency modulated ultrasound (20–40 kHz) was applied to AlSi10Mg. By dynamically switching the frequency multiple times per second to match system resonance, standing wave patterns under fixed-frequency conditions were effectively disrupted, preventing uneven acoustic fields in nodal regions of the melt pool. This technique significantly improved the uniformity of acoustic energy distribution in large, complex geometries—such as multi-layered aerospace turbine casings—promoting uniform cavitation throughout the melt pool. Grain sizes were refined to ~15–20 μm, the proportion of columnar grains was reduced to <20%, and hydrogen content was decreased by >50%. The method is particularly suitable for non-planar, complex parts, ensuring microstructural consistency, increasing overall fatigue life by 30%, and providing reliable control for industrial-scale production [58].

### 3.5. Solidification Defects During AM

Solidification defects such as shrinkage porosity, hot cracking, liquation cracking and lack-of-fusion voids remain a major bottleneck in metal AM, severely limiting the mechanical performance and reliability of printed components. These defects arise from the highly non-equilibrium solidification conditions inherent to laser- or electron-beam-based processes: thermal gradients typically reach 10^5^–10^7^ K m^−1^, while cooling rates often exceed 10^3^–10^6^ K s^−1^. Such extreme conditions drive rapid solid–liquid interface advancement, severe solute trapping, complex melt-pool hydrodynamics, and highly nonlinear defect-formation mechanisms that are challenging to predict and control precisely. Recent XCT characterization studies have demonstrated that UF-LBAM can substantially suppress porosity formation under extreme non-equilibrium conditions, highlighting its considerable potential for regulating such defects [59].

**Figure 6:**
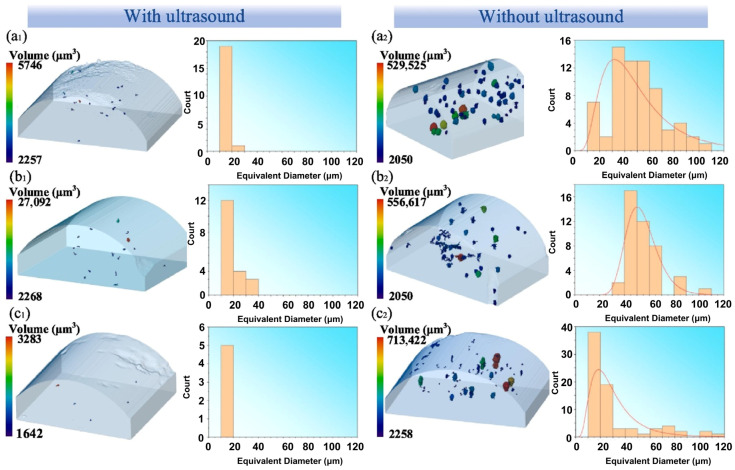
CT reconstructions of pore defects under three laser parameters: (**a1**,**a2**) Laser power of 1000 W with scanning speed of 10 mm/s; (**b1**,**b2**) laser power of 1400 W with scanning speed of 4 mm/s; (**c1**,**c2**) laser power of 1400 W with scanning speed of 10 mm/s [59]. The red line represents the fitted result.

Figure 6 presents 3D reconstructed CT scans of clad layers produced under varying laser process parameters, both with and without applied ultrasound. Quantitative analysis reveals that ultrasonic assistance markedly reduces pore population and total volume in clad layers: large pores with equivalent diameters > 40 µm are essentially eliminated, while the number of pores in the 20–40 µm range decreases substantially. The average equivalent pore diameter drops from ~45 µm to ~18 µm, and overall porosity fraction decreases from ~0.28% to ~0.11%. Mechanistic insights indicate that ultrasonic vibration significantly enhances melt-pool flow velocity, promoting bubble flotation, coalescence, and escape under the combined action of convective currents and acoustic streaming, thereby effectively inhibiting pore entrapment and retention. Furthermore, Figure 6a,c illustrate that, within an appropriate process window, increasing laser power further optimizes the thermal–hydrodynamic coupling in the melt pool, yielding additional porosity reduction. The pronounced decline in porosity directly mitigates local stress concentration, suppresses crack initiation and early propagation, prevents premature clad-layer failure, and ultimately enhances the overall mechanical properties and service reliability of AM components.

## 4. Applications for Machine Learning in Manufacturing

Machine learning (ML) not only serves as a tool for feature recognition and quantitative analysis of X-ray images, but has also emerged as a powerful approach for real-time monitoring of product quality and defect detection during LBAM [27,60,61]. ML has gradually been applied to thermal imaging monitoring since the mid-2010s, and particularly after 2018, ML algorithms (e.g., CNNs) have become mainstream tools for fault detection, predictive maintenance, and anomaly monitoring. Representative ML algorithms used in LBAM are summarized in Table 2.

**Figure 7:**
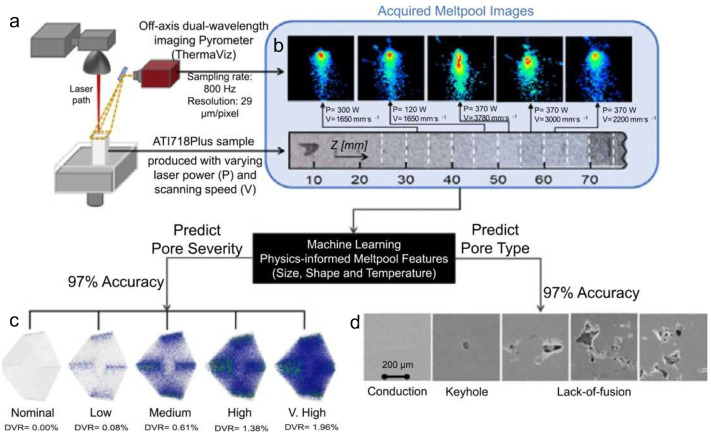
(**a**) Schematic representation of the experimental setup; (**b**) representative melt pool images acquired in situ; (**c**,**d**) combined X-ray computed tomography (XCT) and scanning electron microscopy (SEM) of XZ cross-sections, illustrating the effect of varying laser power on microstructural features [73].

Integrating ML into defect-detection systems yields deeper mechanistic insights into LBAM processes by revealing hidden correlations in multimodal sensor data. This capability arises from ML’s proficiency in identifying implicit patterns and establishing nonlinear mappings between process parameters—such as laser power, scan speed and powder feed rate—and final part quality, including density, surface finish and mechanical performance [62]. As a core subset of artificial intelligence (AI), ML equips systems with the ability to learn directly from data and progressively enhance performance without explicit programming. A wide array of ML models has already been developed and deployed for defect detection across AM modalities [63]. The present discussion centers on algorithms tailored specifically for real-time defect identification in metal LBAM, where rapid decision-making is essential to mitigate issues such as porosity, cracking and lack-of-fusion [64,65]. ML approaches are broadly categorized into four primary paradigms (Figure 7): (1) supervised learning, which relies on labeled datasets to train models for classification or regression tasks [66]; (2) unsupervised learning, which identifies intrinsic structures and anomalies in unlabeled data [67]; (3) semi-supervised learning, which leverages limited labelled data alongside abundant unlabeled data to improve generalization [68]; and (4) reinforcement learning, which optimizes sequential decision-making through trial-and-error interactions with the process environment [69]. Each paradigm offers unique advantages for LBAM defect detection—supervised methods excel in well-characterized defect classification, unsupervised techniques enable anomaly discovery in novel conditions, semi-supervised approaches address data-scarcity challenges, and reinforcement strategies support adaptive process control—as elaborated in the subsequent sections with illustrative applications.

**Table 2:** Machine learning algorithm in LBAM [70,71].

Machine Learning Algorithm	Definition	Application Scenarios	Advantages	Disadvantages
Supervised Learning	Uses large amounts of labeled data to train models and establish direct input → output mappings (classification, regression)	- Image-based defect detection (porosity/cracks/spatter with CNN/YOLO/U-Net) - Process parameter–performance prediction (tensile strength, hardness) - Melt pool geometry regression	Highest accuracy (95–99% common); directly usable for classification/prediction; most mature for industrial deployment	Extremely dependent on high-quality labeled data (metal printing data is expensive, labeling cost is very high); poor generalization (fails easily when changing materials/machines)
Unsupervised Learning	No labeled data—automatically discovers inherent data structures (clustering, dimensionality reduction, anomaly detection)	- Anomaly detection (Autoencoder/GAN discovers unknown defects without any defect labels) - Powder spreading/melt pool image clustering - Data dimensionality reduction and feature mining	No labeling required—ideal for data-scarce scenarios; discovers “unknown” defects; reduces labor cost	Usually lower accuracy than supervised learning; results have poor interpretability; difficult to use directly for precise classification/prediction
Semi-supervised Learning	Combines a small amount of labeled + large amount of unlabeled data (consistency regularization, self-training, etc.)	- Few-shot defect detection (only label a small number of images, use the rest of unlabeled video for training) - Cross-machine/material transfer learning (leveraging massive unlabeled process data) - GAN-based data augmentation	Dramatically reduces labeling cost (especially practical in metal printing); accuracy close to fully supervised; stronger generalization	Performance heavily depends on the quality of the few labeled samples; training is more complex (needs pseudo-label mechanisms); risk of introducing noise
Reinforcement Learning	An agent learns the optimal policy through trial and error + reward feedback interacting with the environment (no supervised labels)	- Closed-loop process control (real-time adjustment of laser power/scanning speed based on melt pool image feedback) - Adaptive parameter optimization (simulator + real-machine joint training) - Sequential decision-making in structural topology optimization	True “adaptive” control; no need for large historical labeled data; handles dynamic uncertainty well	Training is extremely slow and unstable (requires massive simulation interactions); high safety risk in real deployment (initial policy may damage equipment); reward function design is difficult

### 4.1. Melt-Pool State Classification and Defect Detection

**Figure 8:**
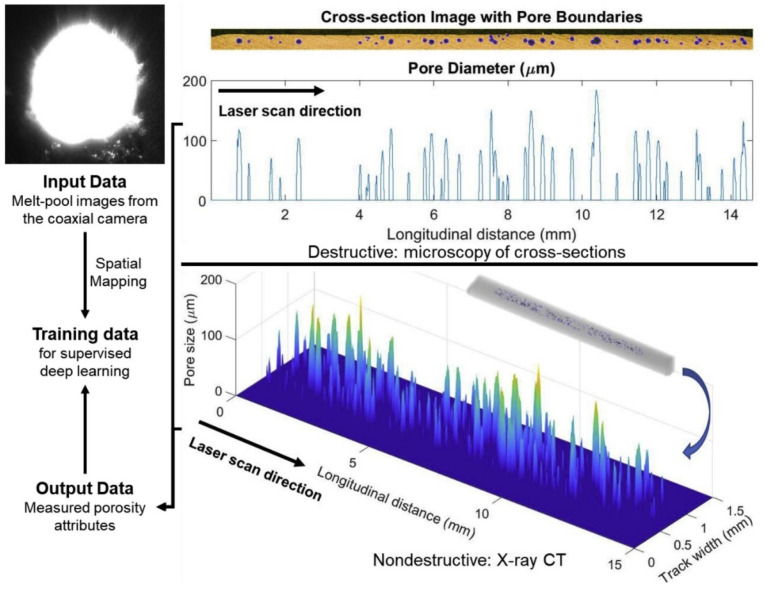
Illustration of quality inspection and input–output data preparation. **Left**: coaxial melt-pool image; **Right top**: pore-level information from a destructive cross section; **Right bottom**: pore-level information from nondestructive X-ray CT [77].

Here, we highlight two representative examples of ML applications in LBAM for Defect detection. The first focuses on supervised ML using the widely adopted k-nearest neighbor (kNN) classification algorithm. Such kNN algorithm [72] is a supervised machine learning method used for classification and regression. It assigns a class to a new data point based on the majority class among its k closest neighbors in the feature space, as determined by a chosen distance metric, typically Euclidean distance. The algorithm is non-parametric and simple to implement, making it well-suited for real-time defect detection in additive manufacturing, where patterns in sensor data or imaging features can be directly mapped to known defect categories. Its performance depends on the choice of k and the quality of the feature representation, and it is particularly effective when the decision boundaries between classes are locally defined rather than globally linear. Ziyad Smoqi et al. [73] demonstrated the monitoring and online prediction of porosity formation during LBAM by combining in situ thermographic images of the melt pool with kNN classification algorithm. A sample was fabricated from ATI 718Plus alloy. By systematically varying laser power and scan speed within a single build, the Ziyad Smoqi et al. intentionally induced a range of porosity types—primarily lack-of-fusion and keyhole pores—with differing severities distributed throughout the part. Melt-pool behavior was continuously captured using an in situ dual-wavelength imaging pyrometer integrated into the LBAM system (See Figure 7). From the thermographic images (Figure 7b), three physically interpretable signatures were extracted: (1) melt-pool length; (2) characteristics of the melt-pool temperature distribution; (3) number, size, and spatial distribution of spatter. XCT and scanning electron microscopy (SEM) images were used as input for the KNN model in Figure 8. Such physics-informed features were then used to train computationally lightweight machine learning models, with KNN serving as a case study [74]. The models simultaneously classified porosity type and severity, achieving an F1-score exceeding 95%. For comparison, a CNN [75] was trained directly on the raw melt-pool images, bypassing any hand-crafted feature engineering. This approach yielded comparable performance, with F1-scores ranging from 89% to 97%. These findings indicate that, for porosity prediction in LBAM, pragmatic and physically meaningful melt-pool signatures combined with simple machine learning models can deliver predictive accuracy on par with that of far more computationally intensive deep learning architectures.

The second example illustrates the application of CNNs—a deep learning framework for extracting hierarchical features from images. Convolutional layers detect local patterns, pooling layers reduce spatial dimensions while preserving essential information, and fully connected layers integrate features for classification or regression. By exploiting shared weights and local connectivity, CNNs efficiently learn from high-dimensional image data, enabling accurate defect detection and quality assessment in additive manufacturing. CNNs offer significant advantages in the analysis and interpretation of X-ray images, primarily due to their robust capability in handling spatial hierarchies and local patterns [76]. Through successive convolutional layers, CNNs automatically extract multi-scale feature representations—from low-level edges and textures to high-level semantic concepts—enabling effective detection of subtle density variations, intricate geometric structures, and defect features (such as cracks, voids, foreign inclusions, or pathological abnormalities) even under substantial noise interference. Direct correlations between conventional melt-pool measurements (e.g., surface dimensions, peak temperature, or average intensity) and resulting porosity often exhibit limited fidelity. Porosity formation in LPBF is governed by highly coupled multi-physics phenomena—including hydrodynamics, thermocapillary convection, recoil pressure, phase transitions, and gas entrapment—along with pronounced nonlinearities and cumulative history effects such as interlayer heat accumulation, residual stress, melt-pool oscillations, and instability modes. Surface-derived features from pyrometry or imaging capture only instantaneous, localized conditions at the melt-pool free surface, failing to adequately resolve subsurface flow fields, keyhole dynamics, bubble nucleation and migration, or transient instabilities that ultimately dictate pore entrapment and persistence.

Nonlinear mapping between melt-pool imagery and pore formation, end-to-end CNN architectures have been widely adopted [78]. These models directly learn pore-level attributes from raw high-speed thermographic images or X-ray images. The model implicitly extracts hierarchical representations of defect precursors and subsequently aggregates these to predict macroscopic porosity metrics, including volume fraction, type distribution (lack-of-fusion vs. keyhole), and characteristic pore dimensions.

Prior to supervised model training, precise spatiotemporal pairing of each high-speed camera frame with the corresponding cross-sectional porosity ground truth is essential. To achieve this, a few studies [79,80] have developed comprehensive image-processing pipelines in MATLAB 2025b, which generally include the following steps in Figure 8:

1. Global registration and stitching of serial cross-sections: Successive metallographic or XCT images of transverse sections were aligned at pixel-level accuracy using the Speeded-Up Robust Features (SURF) algorithm [81] for feature detection and matching. This establishes a consistent coordinate frame across the entire build height, enabling accurate mapping of melt-pool observations to their corresponding defect locations.
2. Orientation correction and region-of-interest cropping: Scan tracks exhibiting tilt or offset relative to the image axes were automatically detected and rotated to align with the primary build direction. Known substrate and fixture geometries were then used to crop extraneous regions (e.g., baseplate, support structures, clamping artifacts, and shadows), isolating the relevant melt tracks.
3. Binarization and pore candidate segmentation: Grayscale images were thresholded using Otsu’s method to generate binary masks of dark regions indicative of voids. Connected-component analysis was performed on candidate pores, with geometric descriptors computed for each: solidity (area/filled area), eccentricity, equivalent diameter, perimeter, and aspect ratio. Physically motivated filters were applied to discriminate true metallurgical pores (e.g., gas porosity, lack-of-fusion voids, keyhole collapse pores) from imaging artifacts, polishing scratches, cracks, oxide inclusions, or textural noise (e.g., solidity > 0.7 and eccentricity < 0.85 thresholds to exclude elongated or irregular non-pore features).

Such automated and reproducible workflow generates high-fidelity ground-truth labels, substantially reducing label noise while ensuring precise spatial correspondence between in situ melt-pool images and ex situ porosity characterization. Supported by rigorous image-to-defect registration, the end-to-end deep learning strategy unveils latent causal relationships between transient melt-pool thermography and the resulting pore distributions across the build.

### 4.2. Porosity Prediction

**Figure 9:**
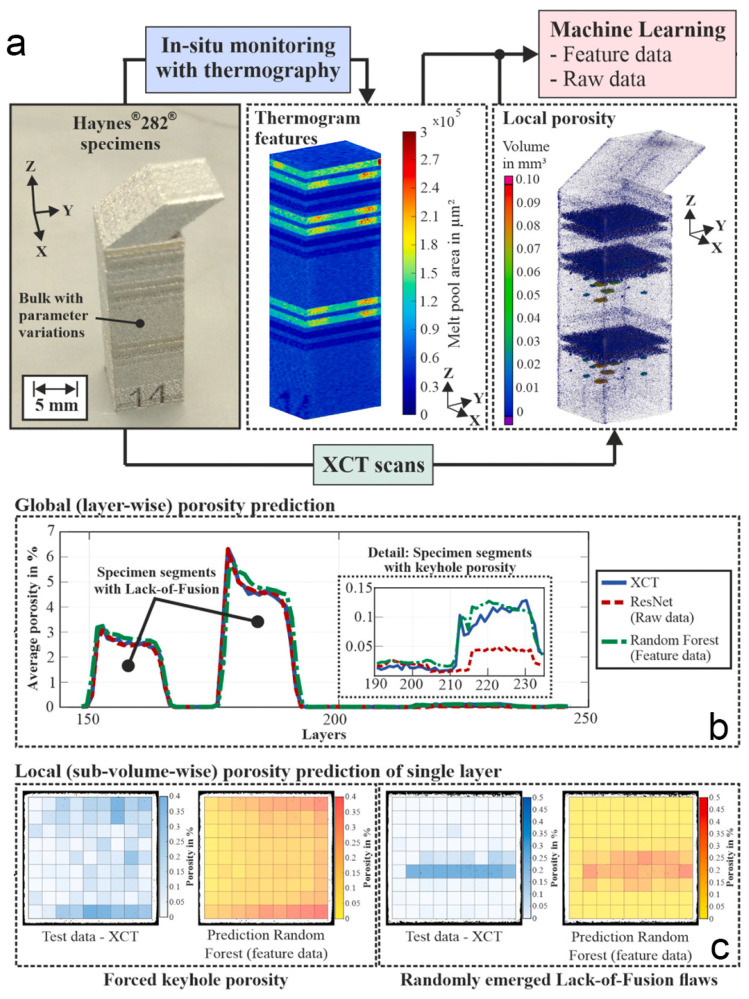
Workflow for porosity prediction in LBAM. (**a**) Input images data, including thermography and XCT scans. (**b**) Global porosity prediction across the entire part. (**c**) Localized, single-layer porosity prediction [84].

The two preceding examples illustrate ML-based feature recognition in LBAM process images, whereas the final case emphasizes its effectiveness in predicting solidification defects [82,83]. It is well known that internal porosity and other defects in parts produced by LBAM severely limit their industrial applicability, as pores can compromise mechanical performance and lead to part failure. Simon Oster et al. investigated the feasibility and effectiveness of in situ thermography, particularly short-wave infrared imaging, for detecting and predicting porosity during manufacturing. For example, to enable local porosity prediction, the feature dataset is organized into ‘clusters’—small-scale rectangular prismatic volumes extracted from the overall specimen (Figure 9a). All feature values within each cluster are averaged and assigned to the corresponding cluster center. For every cluster, a corresponding porosity label is computed, which requires precise registration of the cluster spatial coordinates with the XCT coordinate system, thereby enabling calculation of the associated porosity. Although feature extraction is a well-established approach that leverages expert knowledge to compress large-scale data into representative features, this process can introduce prior bias into the modeling and, because the model input comprises only a limited number of hand-crafted features; potentially important spatiotemporal information present in the raw data is often underutilized.

Accordingly, in addition to feature-based approaches, porosity prediction can be performed using a regression model that operates directly on raw image sequences, denoted here as IMSEQR. This model takes pre-processed melt-pool image sequences as input and can automatically learn the intrinsic relationships between temporal melt-pool evolution and defect formation without relying on manual feature selection. Dataset construction follows two principal guidelines: first, to maintain computational cost within reasonable bounds, the scale of the raw image sequences is constrained by cropping a region of interest (ROI) around the melt-pool center in each frame, thereby reducing image dimensionality; second, to ensure comparability of the sequence modeling results with those of the feature-based approach, the image sequences are divided into cluster-specific subsequences, retaining only those frames in which the melt-pool center lies within the boundaries of the corresponding cluster. A maximum of 25 frames is retained per cluster, with excess frames discarded; when fewer than 25 frames are available, zero-frame padding is applied to maintain constant sequence length. In addition, timestamp information for each frame is preserved in the sequence. The predicted porosity, both at the global and local scales, is presented in Figure 9b,c. Such capability is demonstrated not only for flaw formation induced by deliberate process parameter variations, but also for randomly formed defects within the specimen bulk.

### 4.3. Microstructures Prediction

**Figure 10:**
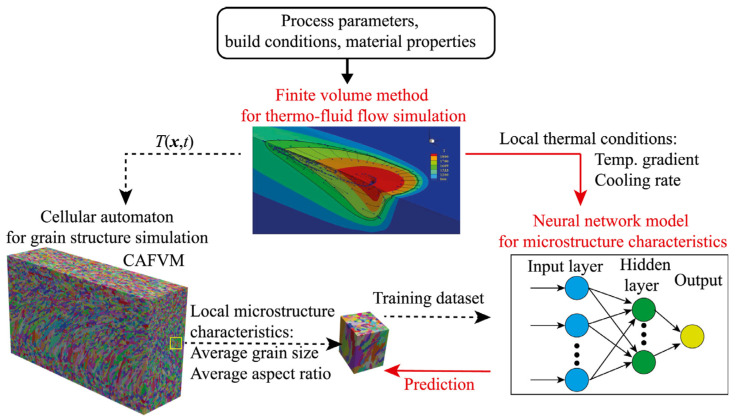
Schematic diagram of the machine learning-based grain structure prediction method to link the local process-related thermal characteristics to the corresponding local microstructure characteristics [85].

To fully leverage the potential of machine learning in metal AM while reducing reliance on “big data,” Kats et al. [85] proposed a physics-informed neural network (PINN) framework. In this framework, data-driven methods are integrated with fundamental physical laws, including the conservation of momentum, mass, and energy, to constrain and guide the learning process. Building on this, a hard-type approach for Dirichlet boundary conditions based on the Heaviside function has been introduced, enabling strict enforcement of boundary conditions and accelerating network convergence. Studies have shown that, due to the incorporation of additional physical prior knowledge, the PINN can accurately predict the temperature distribution and melt pool dynamics in metal AM processes even with only a moderate amount of labeled data. As illustrated in Figure 10, the ML-based microstructure prediction method is divided into three stages. First, the finite volume method (FVM) is used to simulate the thermo-fluid field under given process parameters, build conditions, and material properties. Second, based on the thermal field obtained from FVM, a three-dimensional cellular automaton (3D CA) method is used to simulate the evolution of the grain structure. Finally, the trained neural network model is coupled with FVM to establish a mapping between thermal conditions (such as temperature gradients and cooling rates) and local microstructural characteristics (such as grain size and aspect ratio). By coupling CA and FVM (CAFVM) to generate the training and testing dataset, this approach enables effective prediction of microstructural features under given directed energy deposition (DED) process parameters. Furthermore, Li et al. [86] introduced a transfer learning-based framework for microstructure reconstruction and structure–property prediction, enabling predictions of new microstructures from existing material datasets. This approach combines an encoder–decoder architecture with feature-matching optimization via a deep convolutional network. In reconstructing microstructures, model pruning is used to probe the relationship between microstructural features and hierarchical layers of the network. Insights gained from pruning are subsequently applied to guide the design and initialization of a structure–property predictive model. Numerical simulations demonstrate that the framework generalizes across a wide range of material microstructures with varying geometric complexity.

### 4.4. Optimized Process Monitoring and Control

At the industrial scale, X-ray imaging offers unparalleled insight into melt pool dynamics and microstructural evolution but is impractical for factory deployment due to its size and complexity. Multi-modal sensor fusion has been demonstrated across several systems to enable industrially viable, real-time process monitoring [87]. For AlSi10Mg in LPBF, in situ X-ray imaging has been used to label pore formation and melt pool disturbances, enabling acoustic emission (AE) sensors to predict defects with over 70% accuracy in complex, multilayer geometries [88]. In direct energy deposition (DED) of Ti-6Al-4V, X-ray data were combined with near-infrared (NIR) thermography and AE, allowing thermal and acoustic signals to be correlated with microstructural evolution, improving particle deposition uniformity by more than 70% and reducing residual stress by ~25% in intricate 3D features such as internal channels or porous implants [89]. High-power industrial-scale AM of Al and Ni alloys has employed X-rays, high-speed imaging, and AE for training models capable of defect prediction without continuous X-ray monitoring, enabling early warning and quality control for large, complex parts. Similarly, biomedical Ti implants with porous architectures have benefited from X-ray guided NIR training, ensuring uniform pore distribution and mechanical consistency while providing real-time feedback during fabrication. These examples illustrate how multi-modal sensor fusion can translate high-fidelity X-ray insights into practical, deployable monitoring strategies for industrial AM.

In constructing ML datasets for industrial defect detection and microstructural analysis, reliable ground-truth annotations are typically obtained from high-fidelity non-destructive characterization techniques [90]. For example, XCT enables the three-dimensional reconstruction of internal structures, while metallographic analysis provides high-resolution microstructural images. These techniques allow the precise identification of defect features such as pores, cracks, and inclusions, including their size, spatial distribution, and morphology. However, the annotation process remains challenging in practice. Annotation noise can arise from subjective variations in manual labeling, resolution limitations of imaging systems, or reconstruction artifacts in XCT data. In addition, domain shift often occurs between laboratory-acquired datasets—typically collected under controlled conditions with uniform illumination—and industrial production data, where variations in lighting, vibration, and material batches lead to distribution mismatches that significantly degrade model generalization performance. Several strategies have been proposed to mitigate these challenges. Transfer learning reduces the dependence on large labeled datasets by pretraining models on large-scale datasets (e.g., ImageNet or domain-specific materials datasets) followed by fine-tuning in the target domain. Domain adaptation methods, including adversarial domain alignment and distribution-matching approaches based on maximum mean discrepancy (MMD), explicitly minimize feature discrepancies between source and target domains, enabling unsupervised or semi-supervised cross-domain transfer. In addition, self-supervised learning leverages large volumes of unlabeled industrial data to learn generalizable feature representations through tasks such as contrastive learning (e.g., SimCLR) or masked reconstruction. These approaches improve robustness to noisy labels and domain shifts. When combined, they can substantially reduce annotation costs—often by 30–70%—while improving cross-domain prediction accuracy by more than 15% [91].

Beyond algorithm development, practical deployment requires careful consideration of engineering constraints. Inference latency must typically remain within milliseconds (often <50 ms) to meet real-time inspection requirements on production lines [92]. This is commonly achieved through model compression techniques such as quantization, pruning, and hardware-specific optimization frameworks (e.g., TensorRT). Sensor synchronization is another critical factor in multi-modal monitoring systems, requiring accurate time alignment between vision cameras, auxiliary sensing probes, and production-line programmable logic controllers (PLCs) to avoid decision errors caused by timestamp drift. Furthermore, computing resources are often limited in edge devices, where power consumption constraints (e.g., <30 W) necessitate trade-offs between model accuracy and computational efficiency. Lightweight architectures, such as MobileNet or EfficientNet variants, are therefore commonly preferred. Finally, model outputs must be seamlessly integrated into industrial feedback and control systems—such as closed-loop PID controllers or manufacturing execution systems (MES)—through standardized communication protocols (e.g., APIs or OPC UA). This integration enables real-time triggering of alarms, parameter adjustments, or automated sorting, forming an end-to-end intelligent quality control loop capable of reliable operation under continuous 24/7 production environments.

## 5. Outlook

The roadmap and near-term challenges of UF-LBAM can be distilled into four critical domains: (1) physics gaps, (2) diagnostics limitations, (3) ML application challenges, and (4) industrialization. Despite transformative advances enabled by synchrotron X-ray imaging, a fundamental understanding of microscale melt flow and acoustic mechanisms across diverse alloys remains incomplete. In particular, the influence of ultrasonic frequency and power on dendrite fragmentation and grain nucleation is not fully resolved, and its universality across alloy systems remains uncertain. High-resolution in situ techniques, including ultrafast synchrotron imaging and XCT, offer unprecedented quantitative insight into melt pool dynamics and microstructural evolution; yet, translation to industrial settings remains limited. Addressing these challenges demands high-resolution multimodal sensor fusion, standardized benchmark datasets, and rigorously designed experimental protocols. ML holds considerable promise for real-time defect detection, porosity prediction, and multi-parameter optimization, but fully realizing this potential requires physics-informed, uncertainty-aware models capable of generalization across alloys and processing conditions, leveraging high-fidelity X-ray datasets for predictive and adaptive microstructure control. Integrating UF-LBAM with ML and high-resolution monitoring unlocks closed-loop control of melt pool dynamics, keyhole stability, and microstructure evolution, dramatically mitigating defects. Future endeavors must prioritize scalable industrial deployment, robust predictive frameworks, standardized protocols, and demonstrable closed-loop control to enable reliable fabrication of high-performance metal components.

## Figures and Tables

**Table 1:** Physical phenomena in LBAM [21,36,37,38,39,45,46].

Phenomenon	Observation Description	Synchrotron X-Ray Parameters	Alloy Systems
Keyhole	Transition threshold from conduction to keyhole mode (power density ≈ 0.4 MW/cm^2^); radial/axial fluctuations (frequency 2.5–10 kHz); rear-wall bulge, collapse and bubble pinch-off; keyhole depth/morphology changes linearly with laser power–speed	Frame rate 50 kHz~MHz, Spatial resolution 1.96 μm	Ti-6Al-4V, Al7075, Copper
Melt Flow	Marangoni convection drives melt-pool circulation (velocity 0.4–2.4 m/s); bubble/porosity migration with flow; melt-pool oscillation and surface waves; competition between acoustic streaming and thermocapillary forces	Frame rate 50 kHz, Spatial resolution 1.96 μm	Invar 36 (Fe-Ni), Al7075, Ti-6Al-4V
Cavitation bubble dynamics	Ultrasound-induced cavitation bubble nucleation, growth, oscillation and collapse (20.2 kHz source); increased bubble density and migration to surface for degassing; suppresses keyhole depth fluctuation and eliminates tip pinch-off porosity	Frame rate 271.6 kHz, Spatial resolution 1.96 μm	Bi-8% Zn alloy, Al6061
Solidification Front	Bubbles/porosity captured, deformed and twisted by advancing solidification front; hydrogen diffusion + solidification shrinkage forms stable pores; bubble–dendrite/cellular front interaction leads to “frozen” defects.	Frame rate 10~20 kHz, Spatial resolution 1.96 μm	Al7075, Ti-6Al-4V

## Data Availability

No new data were created or analyzed in this study. Data sharing is not applicable to this article.
